# Novel imaging techniques to study postmortem human fetal anatomy: a systematic review on microfocus-CT and ultra-high-field MRI

**DOI:** 10.1007/s00330-019-06543-8

**Published:** 2019-12-13

**Authors:** Y. Dawood, G. J. Strijkers, J. Limpens, R. J. Oostra, B. S. de Bakker

**Affiliations:** 1 Obstetrics and Gynaecology, Amsterdam UMC, University of Amsterdam, Meibergdreef 9, Amsterdam, The Netherlands; 2 Medical Biology, Section Clinical Anatomy and Embryology, Amsterdam UMC, University of Amsterdam, Meibergdreef 9, Amsterdam, The Netherlands; 3 Biomedical Engineering and Physics, Amsterdam UMC, University of Amsterdam, Meibergdreef 9, Amsterdam, The Netherlands; 4 Research Support - Medical Library, Amsterdam UMC, University of Amsterdam, Meibergdreef 9, Amsterdam, The Netherlands

**Keywords:** Fetus, Anatomy, Magnetic resonance imaging, X-ray microtomography, Contrast media

## Abstract

**Background:**

MRI and CT have been extensively used to study fetal anatomy for research and diagnostic purposes, enabling minimally invasive autopsy and giving insight in human fetal development. Novel (contrast-enhanced) microfocus CT (micro-CT) and ultra-high-field (≥ 7.0 T) MRI (UHF-MRI) techniques now enable micron-level resolution that combats the disadvantages of low-field MRI and conventional CT. Thereby, they might be suitable to study fetal anatomy in high detail and, in time, contribute to the postmortem diagnosis of fetal conditions.

**Objectives:**

(1) To systematically examine the usability of micro-CT and UHF-MRI to study postmortem human fetal anatomy, and (2) to analyze factors that govern success at each step of the specimen preparation and imaging.

**Method:**

MEDLINE and EMBASE were systematically searched to identify publications on fetal imaging by micro-CT or UHF-MRI. Scanning protocols were summarized and best practices concerning specimen preparation and imaging were enumerated.

**Results:**

Thirty-two publications reporting on micro-CT and UHF-MRI were included. The majority of the publications focused on imaging organs separately and seven publications focused on whole body imaging, demonstrating the possibility of visualization of small anatomical structures with a resolution well below 100 μm. When imaging soft tissues by micro-CT, the fetus should be stained by immersion in Lugol’s staining solution.

**Conclusion:**

Micro-CT and UHF-MRI are both excellent imaging techniques to provide detailed images of gross anatomy of human fetuses. The present study offers an overview of the current best practices when using micro-CT and/or UHF-MRI to study fetal anatomy for clinical and research purposes.

**Key Points:**

• *Micro-CT and UHF-MRI can both be used to study postmortem human fetal anatomy for clinical and research purposes.*

• *Micro-CT enables high-resolution imaging of fetal specimens in relatively short scanning time. However, tissue staining using a contrast solution is necessary to enable soft-tissue visualization.*

• *UHF-MRI enables high-resolution imaging of fetal specimens, without the necessity of prior staining, but with the drawback of long scanning time.*

**Electronic supplementary material:**

The online version of this article (10.1007/s00330-019-06543-8) contains supplementary material, which is available to authorized users.

## Introduction

Magnetic resonance imaging (MRI) and computed tomography (CT) have been extensively used to study fetal anatomy for research and diagnostic purposes, enabling minimally invasive autopsy and giving insight in human fetal development [1–4]. However, postmortem MR imaging using clinical routine magnets (1.5–3 Tesla (T)) demonstrates a low diagnostic accuracy, especially in fetuses below 20 weeks of gestation [5–7], as result of low resolution in small samples. Conventional postmortem CT without a contrast agent even shows a lower detection rate for major structures in comparison to MRI [8], not only because of the limited resolution but predominantly due to poor soft-tissue contrast. Novel (contrast-enhanced) microfocus CT (micro-CT) and ultra-high-field (UHF; 7.0 T and higher) MRI (UHF-MRI) techniques now enable micron-level resolution [9, 10], which overcomes the disadvantages of low-field MRI and conventional CT.

Micro-CT is an emerging imaging tool within the biomedical field, which has been developed to scan small samples on high resolution. Its technology is based on X-ray attenuation, just like conventional CT, although with some construction differences. In most micro-CT systems, the radiation source is fixed with the sample mounted on an adjustable and rotating platform. This allows for the adjustment of the “radiation source-to-sample” and “sample-to-detector” distance, giving improved resolution [11]. The addition of contrast, often referred to as *staining*, enables high-resolution imaging of soft tissue. This feature has already been extensively used in animal research [12]. UHF-MRI is a result of the technological capability of increasing the magnetic field strength that results in a higher signal-to-noise ratio, better spatial resolution, and more detailed imaging of fetal anatomy. This is supported by previous studies that have shown that UHF-MRI is superior to low-field MRI in the detection of anatomic details [13–15].

As the technology advances and scanners become increasingly accessible [16], there is a growing interest in the use of these techniques by radiologists in particular to study fetal anatomy postmortem for different purposes, especially in the field of (forensic) radiology and pathology. In these disciplines, a detailed scan of the fetus can contribute to the assessment of fetal conditions and perhaps eventually may serve as substitute for autopsy to determine the cause of death or to study congenital anomalies [14, 17]. However, the collective result of previous studies on the novel applications of these techniques is a complex landscape of highly varied approaches that differ in specimen preparation, staining protocols, CT/MRI hardware, and imaging parameters. A comprehensive evaluation and assessment of the available literature is required to provide practical tools to scan human fetuses by micro-CT and UHF-MRI.

The purpose of the present study is to systematically review the use of micro-CT and/or UHF-MRI to examine postmortem human fetal anatomy. Due to the discussed high variability in imaging approaches, no direct comparison (in the form of a meta-analysis) between these approaches is made. Instead, we will (1) give a comprehensive overview of the usability of these imaging techniques to study fetal anatomy, and (2) analyze factors that govern success at each step of the specimen preparation and imaging based on the range of existing published studies.

## Materials and methods

This systematic review, registered in PROSPERO (number CRD42018092185), followed the Preferred Reporting Items for Systematic Reviews and Meta-analyses (PRISMA) statement [18]. The complete methodology is presented in Electronic Supplementary Material (ESM) 1.

In summary, an experienced information specialist performed a broad search in OVID MEDLINE and OVID EMBASE from 1995 to July 22, 2019, to find studies on fetal imaging by micro-CT or UHF-MRI.

Two authors independently screened all identified publications for eligibility using Rayyan [19]. To avoid inclusion of multiple publications from the same research group using the same database and scanning technique, only one paper was selected based on the most complete method description, followed by the largest number of specimens. The complete dataset including multiple publications is added in Excel as ESM 2.

The following study characteristics were extracted: scanning method, number of specimens scanned, gestational age, anatomical region of interest, staining protocol, scanner model, voxel size, and acquisition time. For micro-CT-focused publications, the following characteristics were also registered: current, voltage, and exposure time. For publications concerning UHF-MRI, the following characteristics were also registered: sequence type, repetition time (TR), echo time (TE), field-of-view (FOV), and matrix size. Corresponding authors were approached if any of the characteristics were missing in the publications.

The results are presented in a descriptive manner, divided per imaging modality and per publication.

## Results

Of the 1831 unique publications identified, 232 were read full text and 39 publications reporting on micro-CT and UHF-MRI were eligible for inclusion in this systematic review (see ESM 3 for the PRISMA flowchart). First, the publications on fetal imaging using micro-CT will be reviewed, followed by the UHF-MRI focused publications. In both cases, best practices concerning specimen preparation (staining) and imaging protocol will be provided.

### Fetal imaging using micro-CT and best practices

A total of 23 unique publications concerned fetal imaging using micro-CT, covering fetal development from 10 to 40 weeks of gestation. The majority of these publications focused on imaging an organ or body part. In four publications, full body scans were conducted of fetal specimens between 10 and 22 weeks of gestation.

#### Staining

**Fig. 1:**
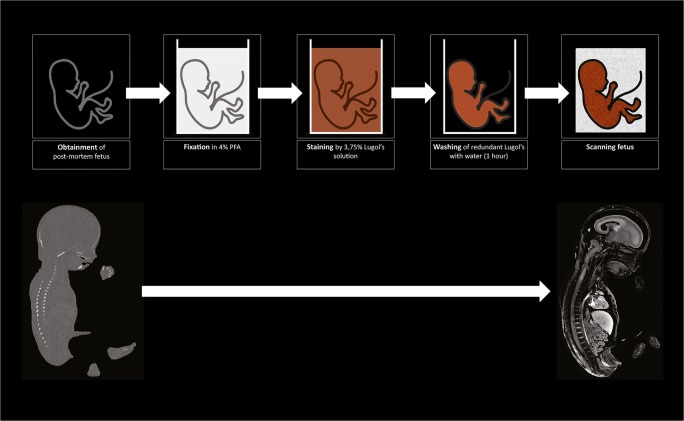
Commonly used workflow of fetus preparation for micro-CT scanning. After obtainment, the fetus is fixed using 4% paraformaldehyde (PFA) or other fixation solution to ensure tissue integrity. This is followed by staining by immersing the fetus in 3.75% Lugol’s solution for days to weeks depending on the fetal size. The solution gives the specimen a typical red-brown color. It is important to wash the fetus after staining to remove redundant Lugol’s solution. Subsequently, the fetus is ready for scanning. Also, on the lower left side, a micro-CT image is shown without staining and on the lower right side a micro-CT image of the same fetus with staining, illustrating the increase of soft-tissue visualization due to staining

Staining is only necessary when a researcher is interested in imaging soft tissue. Without staining, micro-CT provides great spatial resolution for high-density structures (e.g., orbit, humerus, femur) even if the structures are not completely ossified yet [20–33]. Staining is performed by immersing the whole fetus or fetal organ in a staining solution. In Fig. 1, the workflow from the obtainment of tissue towards the scanning including the staining step is depicted.

**Fig. 2:**
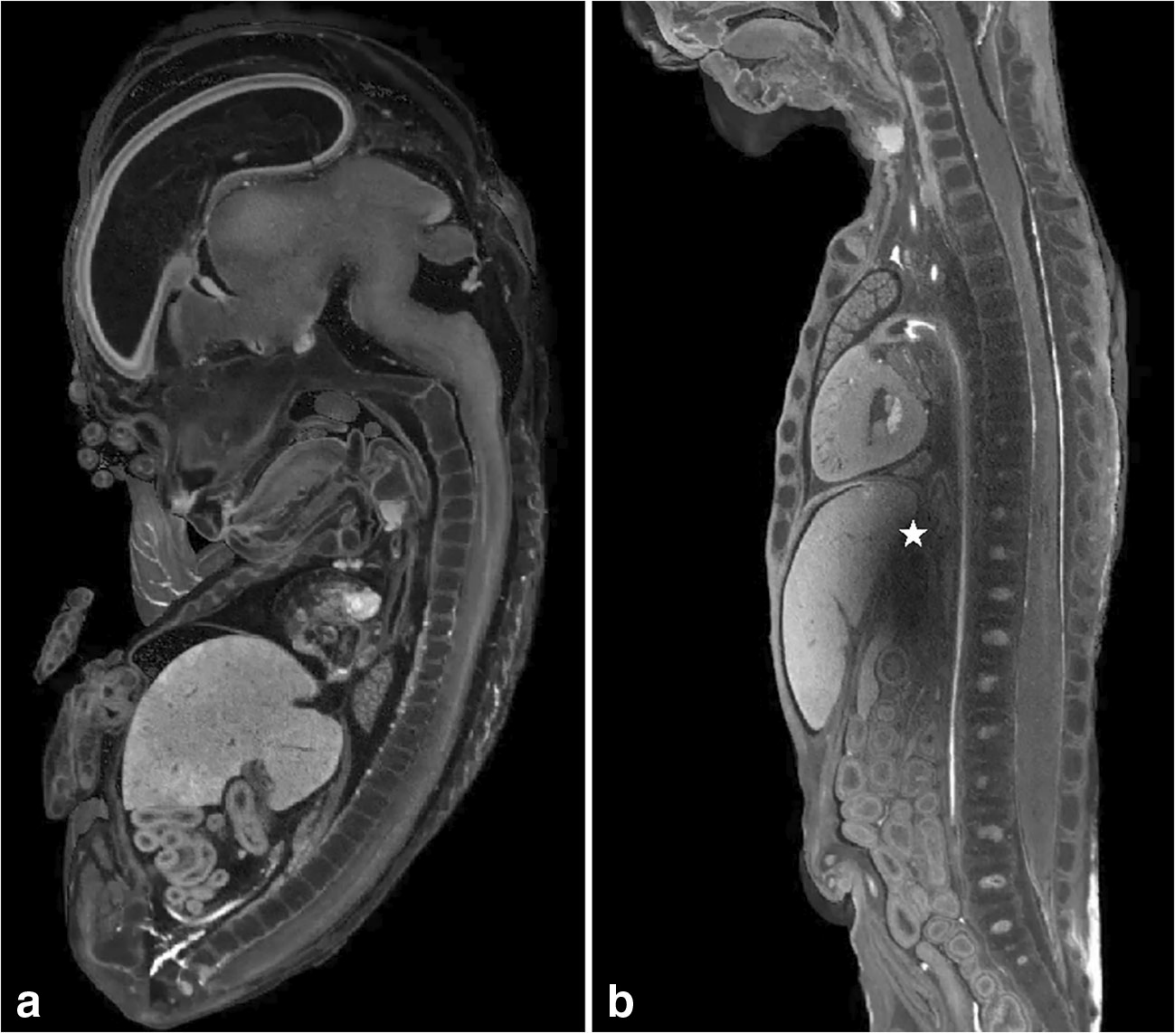
Micro-CT images of two Lugol stained fetuses. **a** Midsagittal view of 11 weeks gestation fetus, 72 h staining with 3.75% Lugol’s solution result in complete and even staining. **b** Midsagittal view of 15 weeks gestation, 72-h staining with 3.75% Lugol’s solution result in artifact (star) as result of incomplete staining. Figure adapted from *Postmortem microfocus computed tomography for early gestation fetuses: a validation study against conventional autopsy (Hutchinson et al. 2018)* [17] with permission from Elsevier (video stills from supplementary data)

For human fetal specimens, the most reported staining solution is a water-based solution containing iodine (I2KI, also called Lugol’s solution, see ESM 4 for more background information, formulations, and protocols). The success of staining depends on three factors: (1) specimen size, (2) staining solution concentration, and (3) staining time. In larger specimens, the staining fluid has to penetrate deeper to reach the core of the fetal body. Staining concentration and time are interdependent factors. A higher concentration results in faster diffusion of the staining solution, enabling shorter staining exposure times. However, extended exposure time (to ensure complete and even staining) with higher concentration can result in overstaining and loss of tissue differentiation and/or tissue shrinkage. The vast majority of the researchers used a 3.75% weight/volume (w/v) Lugol’s solution for a period of 48 h up to 7 days. This concentration is approximately isotonic; therefore, tissue shrinkage as a result of extraction of water should not occur [34]. Spaw and Witmer [35] confirmed that a prolonged staining period of several months in higher concentration (5.5%) did lead to extensive tissue shrinkage, but did not result in better staining of internal structures. Hutchinson et al [17] conclude that 3.75% Lugol for 72 h provides excellent results even for larger specimens (up to 21 weeks of gestation). However, this conclusion is not supported by the supplementary data that accompanied their paper, as a video still of a 15-week-old fetus, stained with 3.75% Lugol for 72 h (Fig. 2), shows an artifact in the liver caused by incomplete diffusion of Lugol. When taking tissue shrinkage and complete and even staining into account, it is therefore recommended to use a 3.75% Lugol’s solution for 48 to 72 h for whole fetal specimens up to 15 weeks of gestation. Fetuses of 15 weeks or older probably need a longer staining time. Furthermore, low-quality, fast scans using a conventional CT scanner can be used to check the progress of staining.

#### Imaging

Specific scanning parameters are dictated to some extent by the hardware and software specifications of the applied imaging system. As can be appreciated from Table 1, several different micro-CT systems have been successfully used by researchers for imaging fetuses. An important issue in choosing a micro-CT system is the size of the specimens intended to scan, as most of the systems can handle full body scanning of fetuses only up to a certain age. For scanning larger specimens, the Nikon XTH ST or XTH 320 and GE Phoenix v|tome|x are suitable since they can handle samples up to 50 kg with a diameter of about 50 cm.

**Table 1:** Imaging protocols reported in the micro-CT imaging literature: *ROI* = region of interest, *%w/v* = concentration in % weight/volume, *NP* = not provided in article and also not retrieved after approaching corresponding author, *PFA* = paraformaldehyde, *EtOH* = ethanol, *PTAH* = phosphotungstic acid hematoxylin

Ref	Number of specimens	Gestational age (weeks)	Anatomical ROI	Fixation protocol		Staining protocol			Scanner model	Current (μA)	Voltage (kV)	(Isotropic) Voxel dimension (μm)	Acquisition time
				Fixation agent	Fixation duration	Stain agent	% w/v	Duration					
Sandrini 2019 [54]	10	13–22	Heart	10% formalin	< 1 year	Lugol	3–3.75	72 h	Skyscan 1176 (Bruker)	264–500	50–89	9–18	NP
Johnson Chacko 2019 [20]	48	17–30	Inner ear	Formaldehyde-glutaraldehyde	Weeks	None			XRadia MicroXCT-400 (Carl Zeiss)	110	45	4.7–14.5	NP
Katsube 2019 [55]	21	15–20	Craniofacial	NP	Years	None			Toscaner-30000 (Toshiba)	180–200	120	35–66	NP
Kramer 2019 [26]	6	22–42	Craniofacial	NP	Years	None			XTH 225 L (Nikon)	NP	NP	NP	NP
Lombardi 2019 [56]	12	10–26	Brain	4% PFA	15–45 days	Lugol	3.75–9	48–120 h	Skyscan 1176 (Bruker)	313	80	18	6–20 min
Schanandore 2018 [27]	1	20	Full body	Formalin	Years	None			V|tome|x s (GE)	200	100	NP	NP
J.C. Hutchinson 2018 [17]	20	11–22	Full body	10% formalin	72 h	Lugol	3.75	72 h	XTH 225 ST (Nikon)	87–180	80–110	7.4–51	57 min
Meignan 2018 [57]	1	22	Pelvic vascularization	Formalin	Unknown	Unknown	Unknown	Unknown	SkyScan 1076 (Bruker)	NP	NP	36	NP
Richard 2017 [28]	8	17–39	Inner ear	10% formalin	1 week	None			VivaCT-40 (scanco medical)	142–176	45–55	10	40–100 min
E.F. Hutchinson 2017 [29]	NP	30–40	Mandible	NP	NP	None			XTH 225 L (Nikon)	83–100	83–100	23–50	NP
J.C. Hutchinson 2016 [36]	6	17–23	Heart	10% formalin	48 h	Lugol	3.75	48 h	XTH 320 (Nikon)	50–135	85–125	19–31	35–70 min
Wu 2016 [58]	3	35–37	Nasolabial muscle	4% PFA	48 h	Lugol	3.75	7 days	Inveon LG (Siemens)	400	60	10	15–20 min
Acquaah 2015 [30]	NP	24–40	Vertebrae	*		None			XTH 225 (Nikon)	NP	NP	28	NP
Skadorwa 2015 [31]	11	16–27	Fallopian canal	10% formalin	4 months	None			SkyScan 1076 (Bruker)	124	80	9	17–40 min
Dumic-Cule 2014 [32]	15	NP	Optic canal and orbital cavity	*		None			SkyScan 1076 (Bruker)	250	50	18	15 min
Lombardi 2014 [59]	21	10–22	Full body and heart	4% PFA	4–7 days	Lugol	3.75, 7.50	2–7 days	SkyScan 1176 (Bruker)	300, 313	80	9, 18, 35	4, 6, 25 min
Spaw 2014 [35]	2	12, 15	Full body	70% EtOH	> 10 years	Lugol	1.25, 5.5	2 h–7 months	eXplore Locus (GE)	NP	NP	20, 45	NP
Siebert 2013 [46]	11	17–31	Bladder	Formalin	NP	PTAH	NP	days	SkyScan 1076 (Bruker)	150	65	9, 18, 35	5–45 min
Reissis 2012 [33]	38	16–40	Humerus and femur	*		None			Actis 420/600 (BioImaging and research)	140–200	40-60	60 × 60 × 100, 120 × 120 × 100	Hours
Shibata 2009 [22]	3	21, 23, 24	Inner ear	10% formalin	40 years	None			Actis (BioImaging and research)	100	80–180	7 × 7 × 40 12 × 12 × 78	NP
Windisch 2007 [23]	7	25–37	Lower extremity	¥		None			Ray Scan 250 E (GmbH)	NP	NP	17–47	NP
Shibata 2006 [24]	2	36	Cranium	Formalin	40 years	None			NP	190	58	97	30 min
Mccoll 2006 [25]	38	16–36	Iliac bone	*		None			Actis 420/600 (BioImaging and research)	140–200	40–60	40 × 40 × 100 60 × 60 × 100	> 120 min

*No fixation, only maceration to clean soft tissue from the bones; ^¥^no fixation, only balming using Thiel’s method

Radiologists and system operators have to consider that beam energies (current and voltage) have to be much higher when scanning iodine-stained fetuses. It is necessary to generate high-energy x-rays that can penetrate the dense, iodine-stained tissues. However, scanning with high energy can lead to excessive x-ray attenuation, and thus to a lower signal-to-noise ratio. Noise can be reduced by modifying x-ray parameters such as exposure time (the time that x-rays pass through the specimen per frame), multi-frame imaging (average of multiple images of the same frame), and rotation step length. As showed in ESM 2, different combinations of exposure timing, multi-frame averaging, and rotation step length are suitable depending on the specimen scanned. Therefore, we recommend to use similar configuration as described in ESM 2 and adapt to own scanner system and specimen.

**Fig. 3:**
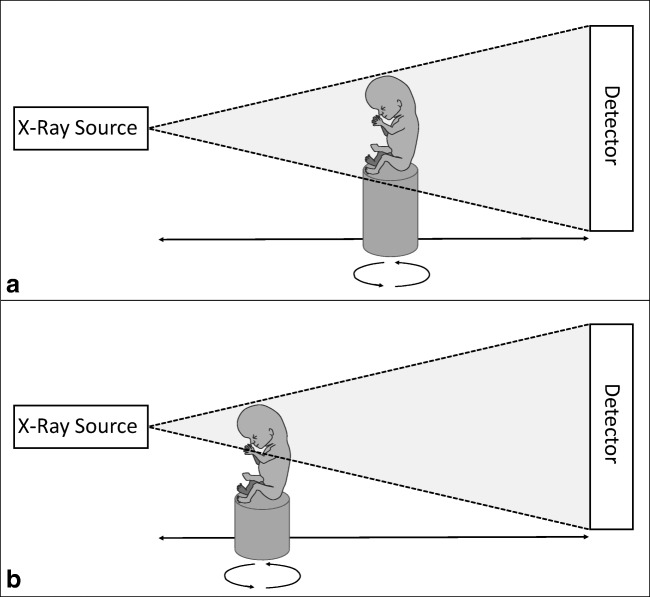
Schematic representation of a micro-CT cabinet setup. The specimen is mounted on a rotating platform and placed between the x-ray source and the detector. The distance between x-ray source and specimen is adjustable enabling scanning the complete specimen at once (**a**), or scanning parts of the specimen resulting in a higher resolution (**b**). When the specimen is scanned in multiple runs, a high-resolution full body dataset can be reconstructed afterwards

**Fig. 4:**
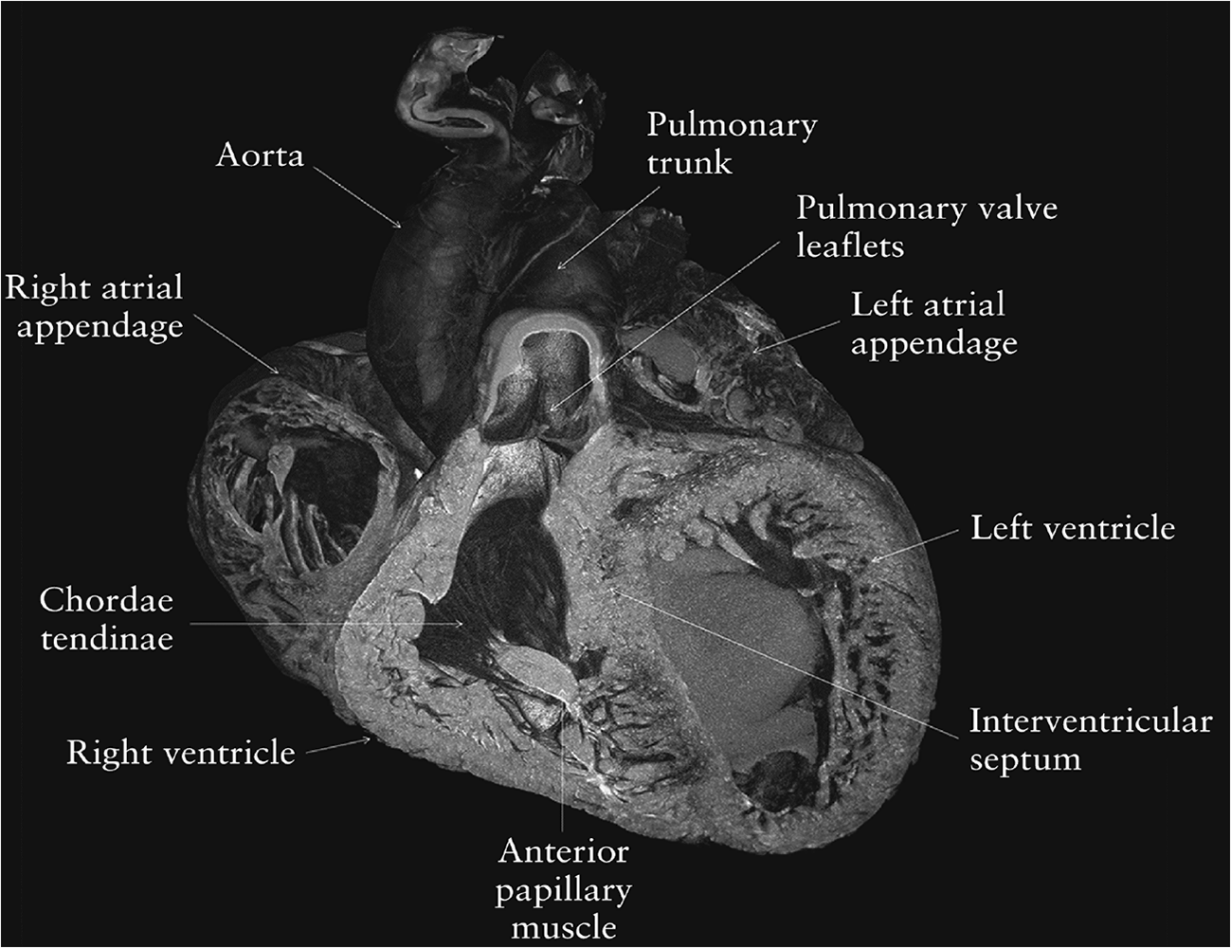
Micro-CT image of a volume rendering of a 23 weeks gestation fetal heart. Cutaway view shows right atrium, left ventricle, interventricular septum, and right ventricular outflow tract with opposed pulmonary valve leaflets. Figure adapted form *Clinical utility of postmortem microcomputed tomography of the fetal heart: diagnostic imaging vs macroscopic dissection (Hutchinson et al. 2016)* [36] with permission from Wiley

Although it is possible to scan fetuses > 20 weeks of gestation, it should be considered that scanning larger samples would mean a loss in image resolution, as resolution is greatly dependent on the relative distance between object and x-ray target. Having the object closer to the x-ray beam would mean (for larger specimens) that it cannot be scanned completely in a single run (Fig. 3). Hutchinson et al [17] resolved this problem by scanning specimens partially in subsequent runs to provide a full body dataset with a resolution of 46 μm of a 21-week-old fetus, which is the oldest human specimen imaged in full using micro-CT published to date. For specific research or diagnostic purposes, it is possible to scan isolated organs, which enables higher resolution and decreases noise from the surrounding tissue (Fig. 4) [36]. As can be appreciated from Table 1, the use of micro-CT systems has enabled researchers to scan full specimens on a resolution of tens of micrometers in just less than an hour. It is recommended to first decide on the desired degree of morphological detail (e.g*.*, only gross anatomy of the major organs, or also nerves and veins) and then on the required resolution. Larger specimens should be scanned in multiple steps or organs should be isolated to reach the desired degree of detail.

### Fetal imaging using UHF-MRI and best practices

A total of 16 unique publications on fetal imaging using ultra-high-field MRI were retrieved, covering the developmental period from 10 to 32 weeks of gestation. The majority of the publications focused on imaging of the brain (8), one on the development of the inner ear and five focused on scanning a body part (e.g., head, thorax) separately. In three publications, full body scans of fetal specimens between 11 and 20 weeks of gestation were conducted.

#### Staining

We could not identify any publications on contrast-enhanced UHF-MRI of human fetal specimens.

#### Imaging

**Fig. 5:**
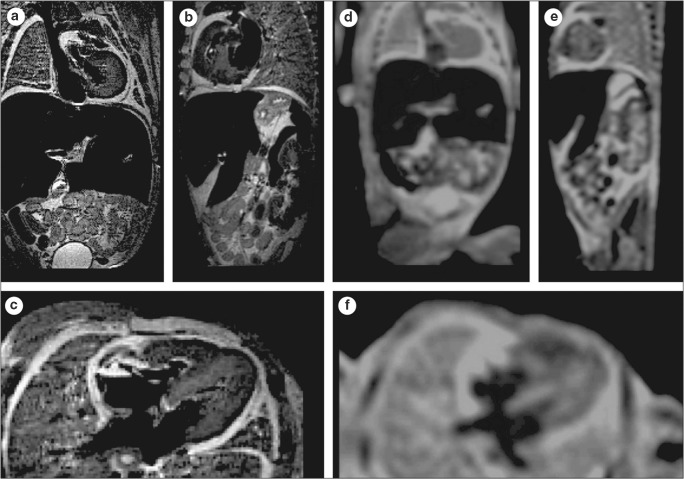
UHF-MRI and low-field MRI images of the same fetus. **a** Coronal, (**b**) sagittal, and (**c**) axial images obtained with three-dimensional T2-weighted MRI at 9.4 T. **d** Coronal, (**e**) sagittal, and (**f**) axial images obtained with three-dimensional T2-weighted MRI at 1.5 T. Figure adapted from *Post-mortem examination of human fetuses: a comparison of whole-body high-field MRI at 9.4 T with conventional MRI and invasive autopsy (Thayyil et al. 2019)* [14] with permission form Elsevier

All examined publications reported successful scanning of human fetuses with a preclinical UHF-MRI scanner (see Table 2) with a field strength from 7 to 11.7 T. Though the bore diameter of most scanners is between the 16 and 30 cm, the use of a radiofrequency (RF) coil limits the inner diameter of the scanning plane to typically less than 10–20 cm, which hampers scanning whole fetuses > 20 weeks of gestation with these preclinical scanners. Several authors examined the added value of UHF-MRI over low-field MRI [14, 15, 37], as demonstrated in Fig. 5 by Thayyil et al (2009) [14]. They conclude, that UHF-MRI enables scanning on much higher resolution resulting in greater diagnostic usefulness, especially in fetuses below 16 weeks of gestation [14, 15, 37].

**Table 2:** Imaging protocols reported in the UHF-MRI imaging literature. *T* = Tesla, *ROI* = region of interest, *TR* = repetition time, *TE* = echo time, *FOV* = field of view, *NP* = not provided in article and also not retrieved after approaching corresponding author, *PFA* = paraformaldehyde. Sequences: *FLASH* = fast low angle shot, *FISP* = fast imaging with steady precession, *RARE* = rapid acquisition with refocused echoes, *DTI* = diffusion tensor imaging, *TSE* = turbo spin echo, *SE* = spin echo

Ref	Field strength (T)	Number of specimens	Gestational age (weeks)	Anatomical ROI	Fixation protocol		Scanner model	Scanning protocol					Voxel dimension (μm)	Acquisition time
					Fixation agent	Fixation duration		Sequence	TR (ms)	TE (ms)	FOV (mm)	Matrix size		
Z. Zhang 2019 [60]	7	60	12–23	Head	10% formalin	< 2 months	Pharmascan 70/16(Bruker)	T1w-RARE T2w-RARE	384 17,000	16 50	60 × 60	256 × 256	NP	NP
Staicu 2019 [61]	7	5	13	Full body	10% formalin	< 1 week	Pharmascan 70/16 (Bruker)	T2w-TSE	3443–5341	36	5 × 3.5	384 × 384	86 × 104 × 500	150 min
Maricic 2019 [62]	7	NP	10–13	Full body	NP	Years	Biospec 70/30 (Bruker)	T1w-FLASH	16.8	8	NP	NP	234 × 235 × 250	NP
Ishikawa 2018 [42]	7	22	10–20	Inner ear	10% formalin	Years	Biospec 70/20 (Bruker)	T1w-FLASH	30	4073–6177	22 × 15 × 15 42 × 28 × 28	636 × 424 × 768 768 × 512 × 512	Isotropic 35–55	20 h
Vulturar 2018 [63]	7	14	15–28	Brain	9% formalin	2 days	Pharmascan 70/16 (Bruker)	T2w-FISP	NP	NP	NP	NP	NP	NP
Krsnik 2017 [41]	11.7	3	10–16	Brain	4% PFA	3–5 days	Bruker	DTI	800	67	25 × 25 × 25 52 × 52 × 52	128 × 80 × 80 128 × 72 × 72	Isotropic 200–400	NP
H. Zhang 2016 [64]	7	45	11–22	Brain	10% formalin	< 1 week	Pharmascan 70/16 (Bruker)	T2w-RARE	17000	50	NP	256 × 256	230 × 230 × 500	28 min
Langner 2016 [65]	7	7	10–12	Upper extremity	4% PFA	NP	ClinScan (Bruker)	T2w-TSE	2000	58	20 × 20	1024 × 1024	20 × 20 × 70	522 min
Ge 2015 [66]	7	41	14–22	Brain	10% formalin	< 2 months	Pharmascan 70/16 (Bruker)	T2w-RARE	12000	50	40 × 40 50 × 50 60 × 60	256 × 256	Slice thickness: 500 In plane: 156–234	NP
Ouyang 2015 [38]	11.7	1	14	Brain	NP	NP	Bruker	DTI	800	66	35 × 28 × 28	128 × 128 × 128	273 × 350 × 350	20 h
Milesi 2014 [67]	7	7	20–32	Hippocampus	4% PFA	4–5 weeks	Biospec 70/30 (Bruker)	T2w-SE	2–8	23–260	NP	NP	80 × 80 × 250	NP
Verhoye 2013 [45]	9.4	9	12–20	Head, thorax, abdomen	Storage in − 20 °C	1 month	Biospec 94/20 (Bruker)	T2w-RARE	2500–7632	33–44	20–50	256 × 256 256 × 128 × 96 256 × 256	NP	1–64 min
Huang 2013 [39]	11.7	4	13–16	Brain	4% PFA	1 week	Biospec 117/16 (Bruker)	DTI	800	67	25 × 25 × 25 52 × 52 × 52	128 × 80 × 80 128 × 72 × 72	Isotropic 200–400	20 h
Zhan 2013 [68]	7	34	15–22	Brain	10% formalin	< 2 months	Pharmascan 70/16 (Bruker)	T2w-RARE	12000	50	NP	256 × 256	190 × 190 × 500 230 × 230 × 500	NP
Votino 2012 [15]	9.4	24	11–20	Heart	Storage in − 20 °C	2–4 months	Biospec 94/20 (Bruker)	T2w-RARE	2500	33, 42.5	20 × 13 × 13 33 × 33 × 33	256 × 128 × 80	80 × 100 × 190, 120 × 210 × 310	64 min
Thayyil 2009 [14]	9.4	17	11–20	Full body	Storage in 4 °C	NP	VNRMS (Varian)	T2w-RARE	500	120	100 × 50 × 50	512 × 256 × 256	Isotropic 200	70 min

As can be appreciated from Table 2, both T1-weighted sequences (e.g., Fast Low Angle Shot, FLASH) and T2-weighted sequences (e.g., Rapid Acquisition with Refocused Echoes, RARE) have been successfully applied for morphological assessment of fetal anatomy in different regions of interest. The diffusion of water in tissues, probed by diffusion tensor imaging (DTI) offers another source of contrast, particularly suited to characterize tissue with a strong structural alignment such as brain white matter. DTI has proven to be a valuable tool in imaging white matter fibers, allowing the study of the formation and orientation of white matter in fetuses [38–41].

Apart from image contrast, imaging resolution is important for the detection and characterization of the smallest anatomical features. Thayyil et al [14] was able to achieve full body datasets with a reasonable resolution of 200-μm isotropic voxel size in 70 min. This is considerably shorter than other groups that needed 20 to 78 h to visualize the development of the inner ear with an isotropic voxel size of 35 to 55 μm [42, 43]. This is due to the fact that reduction in voxel size by a factor 2 in 3-dimension results in a reduction in signal by a factor 2 × 2 × 2 = 8. Maintaining the signal-to-noise ratio (SNR) in the image at this smaller voxel size would require 8 × 8 = 64 times more signal averages (number of excitations) and thus a 64 times longer scan time. As shown in Table 2, several researchers were able to achieve higher resolution than Thayyil et al [14] in shorter scanning time, but they only scanned organs partially with a limited amount of slices and thus were able to use small sensitive RF-coils [15, 44, 45]. As with micro-CT, it is recommended to first decide on the desired degree of morphological detail and contrast, and then on the required resolution. One should be aware of the drawbacks on SNR and thus image quality, as well as the increase in required scan time with decreasing voxel size. Sequences, TE, and TR from Table 2 can be used and adapted to the researchers’ own scanner system and research question.

## Discussion

Micro-CT and UHF-MRI are excellent imaging techniques that have proven to provide detailed images of gross anatomy of smaller animals, human embryos, and human fetuses. The present study offers an overview of the current best practices when using micro-CT and/or UHF-MRI to study human fetal anatomy. As the scope of this review was on imaging human fetal specimens, we have only discussed approaches at each step of preparation and imaging for human fetal specimens. However, publications on imaging of other ex vivo tissues could have reported on other approaches that are equally successful.

### Staining

Several groups showed that immersion in Lugol’s solution is an effective staining technique for human fetal specimens. One group [46] used phosphotungstic acid (PTA), however with an unknown concentration and without clear description of staining time. Other staining techniques (e.g., I2E or I2M, phosphomolybdic acid, and osmium tetroxide) previously used on embryos or animal specimens are not discussed, although they could be equally or even more effective [10, 12, 47, 48]. Future research should address the use of different staining solutions for human fetal imaging.

For specimens older than 15 weeks, submersion in 3.75% Lugol for 72 h is insufficient for complete and even staining. This is in line with earlier research by Li et al [49] on equally sized adult animal specimens, in which 45 days of staining was considered necessary to achieve complete and uniform staining. They also demonstrated that it is necessary to refresh the Lugol’s solution frequently when staining for such a long period. In the case of human fetal specimens, it is unknown if 45 days is really necessary as the epidermis of adult animal specimens is less permeable. Moreover, Li et al [49] used a slightly lower Lugol concentration which adds to a slower staining saturation. This discrepancy between the publications underlines the necessity for appropriate staining protocols specifically for older human specimens.

Further conventional autopsy and histological examination of fetal specimens remains possible after staining when using an iodine-based staining (e.g., Lugol). However, destaining is necessary as the staining solution gives the specimen a red-brown color. Destaining is fairly quick and easy, by completely submerging the specimen in a 4 to 5% weight/volume (w/v) sodium thiosulfate solution for hours to days (see ESM 4 for more background information, formulations, and protocols). It should be noted, however, that destaining does not restore a specimen to its original chemical state, in contrast to what was stated by Hutchinson et al [17]. Rather than being deiodinated, sodium thiosulfate reacts with aqueous triiodide (which is red-brown) reducing it to iodide (which is transparent) and remains in the specimen [12, 50]. If necessary, withdrawal of the iodide is possible with leaching; clean water or fresh storage solution is used to displace iodide due to an osmotic imbalance. However, this process often takes weeks and requires frequent refreshment of the leaching solution, as it becomes saturated with iodide [12].

We could not trace any publications on contrast-enhanced UHF-MRI of human fetal specimens. However, using a gadolinium-based contrast agent could be helpful since the thus-acquired reduction of the T1 relaxation time by the contrast agent allows for a reduction in TR and therefore more signal averages within the same scanning time [9]. We found two publications using a lower field strength MRI and gadolinium as contrast agent on human fetal brains [51, 52]. They removed the brains from the cranium of 22 specimens between 17 and 40 weeks and immersed them in a solution containing 1 mM gadolinium for at least 1 week to reduce the T1 and T2 relaxation times, however without comparison with non-stained samples. Therefore, more research is necessary to investigate the potential gain of gadolinium and other contrast agents prior to imaging, especially concerning whole body fetal specimens.

### Imaging

Although no direct comparison between micro-CT and UHF-MRI is made in any of the papers we included, micro-CT has some advantages compared to UHF-MRI concerning resolution and scanning time; 20 to 78 h of scanning time is necessary to achieve a voxel size of 35–55 μm using UHF-MRI, whereas this can be done in less than an hour by using micro-CT. However, a drawback of micro-CT is that it often requires days of staining to provide soft-tissue contrast, while MRI is suitable for soft-tissue imaging without any preparation. This makes it clinically more convenient for postmortem diagnostics. Furthermore, MRI offers a variety of sequences to address specific clinical or research questions. DTI, for example, could be of value to study (the development of) white matter [40]. A future perspective could be combining both imaging techniques if they yield complementary results, which can be of added value to increase the visualization of various morphological features [53].

**Fig. 6:**
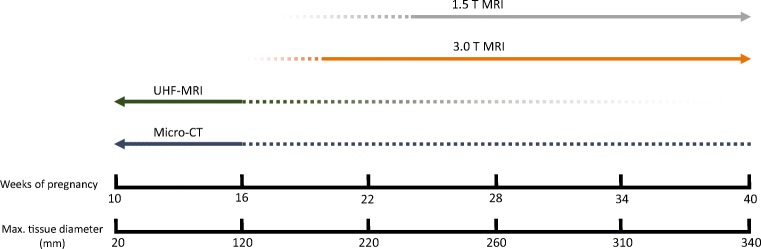
Tool to select the ideal imaging modality for ex vivo diagnostic fetal imaging. MRI with low-field magnets are more suitable for older fetuses from 20 weeks gestation, whereas UHF-MRI and micro-CT are best for fetuses below 16 weeks’ gestation and isolated organs with a maximum tissue diameter below 120 mm due to the small bore size (UHF-MRI) and prolonged staining time (Micro-CT). Older fetuses and tissue samples with larger diameters can still be scanned on selected UHF-MRI devices with larger bore sizes, or by micro-CT after multiple weeks of staining, which delays the autopsy process considerably, making it less suitable in the clinical setting

When considering micro-CT and UHF-MRI for ex vivo diagnostic fetal imaging, they are most suitable for isolated organs or whole body imaging up to 16 weeks’ of gestation (see Fig. 6 for a proposed ideal workflow) due to the following arguments: firstly, the limited bore diameter (for UHF-MRI), secondly the prolonged staining time (for micro-CT), and thirdly the accessibility of the lesser expensive 1.5- and 3.0-T MRIs that have shown comparable diagnostic results with UHF-MRI beyond 20 weeks’ of gestation [15]. Also, as conventional autopsy is typically challenging and therefore bound by limitations in these early first and second trimester fetuses, UHF-MRI and micro-CT can aid by providing morphological data with three-dimensional histological quality in these small specimens [54]. Considering the limited experience with these techniques and their additional costs, conventional autopsy followed by histology will presently remain the gold standard in diagnosing fetal pathology. Nevertheless, as a growing number of centers are gaining access to UHF-MRI and/or micro-CT, we expect them to fulfill a more prominent role in postmortem fetal diagnostics of small samples in due time, provided that future research will not only focus on technical optimization and comparison with conventional diagnostics, but also on normal fetal development in the late first and second trimester, as we are currently lacking organ-specific reference data of the human fetal development.

## Conclusion

Micro-CT and UHF-MRI are both excellent imaging techniques to provide detailed images of gross anatomy of human fetuses. The results of this study offer an overview of the current best practices when using micro-CT and/or UHF-MRI, which we hope will encourage radiologists and other researchers to study fetal anatomy for clinical and research purposes. However, correct assessment of these high-resolution images is not possible without the collaborative effort between trained anatomists, pathologists, radiologists, and other clinicians.

## Electronic supplementary material

ESM 1 (DOCX 38 kb)

ESM 2 (XLSX 29 kb)

ESM 3 (PDF 76 kb)

ESM 4 (PDF 72 kb)

## Abbreviations

DTI: Diffusion tensor imaging
FISP: Fast imaging with steady precession
Micro-CT: Microfocus computed tomography
PTA: Phosphotungstic acid
UHF: Ultra-high field
