# Supplementary material for: Novel imaging techniques to study postmortem human fetal anatomy: a systematic review on microfocus-CT and ultra-high-field MRI
Source: Eur Radiol. 2019 Dec 13;30(4):2280–92. doi: 10.1007/s00330-019-06543-8 (PMC7062658; doi:10.1007/s00330-019-06543-8)
Supplement: Supplementary file 1 — (DOCX 38 kb) [file 330_2019_6543_MOESM1_ESM.docx]

**Materials and Methods**

**Search strategy**

This systematic review, registered in PROSPERO (number CRD42018092185), followed the Preferred Reporting Items for Systematic Reviews and Meta-analyses (PRISMA) statement [1]. An experienced information specialist performed a broad search in OVID MEDLINE (including Epub Ahead of Print, In-Process & Other Non-Indexed Citations) and OVID EMBASE from 1995 to July 22^nd^, 2019 to find studies on fetal imaging by micro-CT or UHF-MRI, using controlled terms (*i.e* MeSH-terms) and text words. Animal studies were safely excluded by double negation. No language or other restrictions were applied. Reference lists and the citing articles of the identified relevant papers were cross-checked in Web of Science. The records retrieved were imported and de-duplicated in ENDNOTE. The complete search strategies are presented in Appendices A and B below.

**Inclusion and exclusion criteria**

The scope of this review was limited to fetal imaging, therefore studies on embryonic and animal imaging were excluded, as these require different imaging protocols, that are not directly applicable to fetal imaging. Fetal age was defined as older than 10 weeks of gestation, based on generally accepted convention [2]. Furthermore, conventional computed tomography or use of MRI with a field strength < 7 T were excluded. Also, reviews, conference abstracts and posters were excluded.

**Study eligibility**

Two authors (YD and BSB) independently screened all identified publications for eligibility using Rayyan [3], first by screening title and abstract and secondly by screening full manuscripts deemed potentially eligible after title and abstract screening. Disagreements were resolved by discussion. To avoid inclusion of multiple publications from the same research group using the same database and scanning technique, only one paper was selected based on the most complete method description, followed by largest number of specimens. The complete dataset including multiple publications is added in Excel as Electronic Supplementary Material 2.

**Data extraction**

Data extraction was performed by one author (YD) using the above-mentioned criteria. If researchers published data on embryonic and fetal tissue, only data concerning fetal tissue was extracted. The following study characteristics were extracted: scanning method, number of specimens scanned, gestational age, anatomical region of interest, staining protocol, scanner model, (isotropic) voxel size and acquisition time. For micro-CT focused publications the following characteristics were also registered: current, voltage and exposure time. For publications concerning UHF-MRI the following characteristics were also registered: sequence type, repetition time (TR), echo time (TE), field-of-view (FOV) and matrix size. Corresponding authors were approached if any of the characteristics were missing in the publications.

**Data analyses**

The results are presented in a descriptive manner, divided per imaging modality and per publication.

**References**

[1] D. Moher, A. Liberati, J. Tetzlaff, D. G. Altman, and T. P. Group, “Preferred Reporting Items for Systematic Reviews and Meta-Analyses: The PRISMA Statement,” *PLOS Med.*, vol. 6, no. 7, p. e1000097, Jul. 2009.

[2] N. J. Klossner, *Introductory Maternity Nursing*. 2005.

[3] M. Ouzzani, H. Hammady, Z. Fedorowicz, and A. Elmagarmid, “Rayyan---a web and mobile app for systematic reviews,” *Syst. Rev.*, vol. 5, no. 1, p. 210, 2016.

Appendix A Medline search strategy

Database(s): **Ovid MEDLINE(R) and Epub Ahead of Print, In-Process & Other Non-Indexed Citations and Daily**1946 to July 19, 2019 
Search Strategy: **2019-07-22**

| **#** | **Searches** | **Results** |
| --- | --- | --- |
| 1 | embryonic structures/ or embryo, mammalian/ or fetus/ or aborted fetus/ or fetal heart/ | 131082 |
| 2 | abortion, spontaneous/ or embryo loss/ or fetal death/ or stillbirth/ or fetal diseases/ or exp abortion, induced/ | 105494 |
| 3 | "embryonic and fetal development"/ or embryonic development/ or fetal development/ | 49212 |
| 4 | exp brain/em or exp neural pathways/em or exp peripheral nervous system/em or exp skeleton/em or exp digestive system/em or exp lung/em or exp kidney/em or exp sense organs/em | 78137 |
| 5 | f?etus*.tw,kf. | 113929 |
| 6 | (f?etal not (f?etal adj3 (blood or sera or serum or osteoblas* or osteocyt* or cord or cells))).tw,kf. | 213495 |
| 7 | (abortion* or stillbirth* or still-birth* or miscarriag* or early pregnancy loss* or (pregnancy adj2 terminat*)).tw,kf. | 86861 |
| 8 | ((human or abort* or normal) adj3 (embryo or embryos)).tw,kf. | 11509 |
| 9 | ((human or normal) adj6 ((embry* adj3 develop*) or embryogenes* or embryo-genes*)).tw,kf. | 5351 |
| 10 | ((early or prenat* or pre-nat* or trimester*) adj2 (developmen* or developing) adj2 (human or embry* or brain* or cereb*)).tw. | 7412 |
| 11 | (neuroembryo* or neuro-embryo*).tw,kf. | 137 |
| **12** | **or/1-11 [ FETUS ]** | **524724** |
| 13 | ((exp animals/ or exp veterinary medicine/ or (animal* or veterin*).jw.) not humans/) or ((nonhuman or experimental model* or animal* or primat* or monkey* or ape or apes or veterinar* or ruminant* or cattle or bovine or cow or cows or horse or horses or mares or equine or sheep or ovine or lamb or lambs or goat* or pig or pigs or piglet* or sow or sows or sus scrofa or swine or swines or porcine or pup or pups or dog or dogs or canine or bitch* or cat or cats or feline or rodent* or lagomorph* or rabbit* or hare or hares or pikas or rat or rats or mice or mouse or murine or serpent* or python*) not human*).ti. or ((knockout* or knock-out* or transgenic* or genetic* modif* or antler* or snake or snakes or chick* or chicken* or shark or sharks or gecko*) not (human or humans)).tw,kf. [animal filter deze nog invoegen] | 5066853 |
| 14 | exp transplantation/ or transplantation, heterologous/ or exp "cell- and tissue-based therapy"/ or exp cells, cultured/ or tr.fs. or (transpl* or allotranspl* or heterotranspl* or homotranspl* or autotranspl* or graft* or allograft* or heterograft* or homograft* or autograft* or cell-therap*).tw,kf. [transplantation] | 2403262 |
| 15 | (cells or in-vitro or cultur*).ti. | 1316745 |
| **16** | **or/13-15 [ animal/transplantation/cell culture exclusion filter ]** | **7212019** |
| **17** | **12 not 16 [ human fetus/embryology/development ]** | **311305** |
| **18** | **limit 17 to yr="1995 -Current"** | **183733** |
| 19 | X-Ray Microtomography/ or ((iodides/ or iodine/) and tomography, x-ray computed/) | 8375 |
| 20 | (microCT* or muCT* or mu-CT* or ((micro or microcomp* or microfocus) adj6 (tomogra* or CT or CTs)) or (microscop* adj (tomogra* or CT*)) or microtomogra* or mutomogra* or mu-tomogra*).tw,kf. | 17516 |
| 21 | (diceCT or dice-CT or (diffus*-Iodin* adj2 contrast-enhanc* adj2 (computed tomograph* or CT or CTs))).tw,kf. | 20 |
| 22 | (PMCT or PMCTs or PM-CT or PM-CTs or ((postmort* or post-mort* or autop* or virtop* or corps* or necrop* or ex-vivo) adj6 (tomogra* or CT or CTs))).tw,kf. | 3016 |
| **23** | **or/19-22 [ I muCT ]** | **22511** |
| 24 | magnetic resonance imaging/ or diffusion magnetic resonance imaging/ or diffusion tensor imaging/ [MRI-mesh] | 398948 |
| 25 | (MRI or MRIs or (magnetic adj2 imag*) or ((MR or NMR or MRM) adj3 (scan* or imag*)) or UHFMRI* or muMRI* or microMRI* or DTMRI or DTI or DWI or diffusion tension imag* or tractograph* or PMMRI* or PMFMRI* or HARDI).tw,kf. | 404409 |
| **26** | **24 or 25 [ MRI-general ]** | **553825** |
| 27 | ((("7" or 7?0* or 7?1 or "8" or 8?0 or "9" or 9?0 or 9?4 or "10" or "11" or "12") adj1 (T or Tesla)) or 7T or 7?0T or 8T or 8?0T or 9T or 9?0T or 9?4T or 11T or 12T or 7TMR or 7?0TMR or 8TMR or 8?0TMR or 9TMT or 9?0TMR or 9?4TMR or 11TMR or 12TMR).tw,kf. | 28761 |
| 28 | (((ultrahigh* or ultra-high*) adj2 field*) or UHF*).tw,kf. | 2448 |
| 29 | (high adj2 field* adj9 (MRI or MRIs or (magnetic adj2 imag*) or ((MR or NMR) adj3 (scan* or imag*)) or DTMRI or DTI or DWI or diffusion tension imag* or tractograph* or PMMRI* or PMFMRI*)).tw,kf. | 2780 |
| 30 | ((high or ultrahigh) adj2 (resol* or strength*) adj3 (MRI or MRIs or (magnetic adj2 imag*) or ((MR or NMR) adj3 (scan* or imag*)) or DTMRI or DTI or DWI or diffusion tension imag* or diffusion imag* or tractograph*)).tw,kf. | 7309 |
| 31 | (super-resolution adj (f?et* or embryo*) adj3 (MRI* or (magnetic adj2 imag*) or (MR adj3 (scan* or imag*)) or DTMRI or DTI or DWI or diffusion tension imag* or tractograph*)).tw,kf. | 1 |
| 32 | (microMR* or muMR* or ((micro or microcomp* or microfocus or microscop* or mu) adj3 (MR or MRI* or magnetic resonan* or NMR or MMR))).tw,kf. | 2683 |
| 33 | (PMMRI* or PMFMRI* or PMMR or ((postmort or post-mort*) adj (scan* or imag*)) or ((PM or PMF) adj3 (MRI or MRIs or resonance imag* or MR or MRM)) or ex-vivo-MRM).tw,kf. | 385 |
| 34 | ((Bruker adj3 (Biospin* or scan* or MR or MRI)) or BioMR-70-20 or PharmaScan* or SIS 200-330).mp. | 141 |
| **35** | **or/27-34 [ UHF or PM MRI ]** | **41140** |
| **36** | **26 and 35 [ II UHF/micro MRI ]** | **15972** |
| 37 | tomography, x-ray computed/ or whole body imaging/ | 367318 |
| 38 | ((CAT-scan* or (comput* adj3 tomogra*)) not (angiogra* or MDCT* or MSCT* or multislic* or multidetect* or dual-energ* or DECT)).tw,kf. | 244896 |
| 39 | ((CT or CTs) adj6 (scan* or imag* or MRI* or tomogra* or whole body or 3D or 3-D)).tw,kf. | 201893 |
| **40** | **or/37-39 [ CT GENERAL ]** | **555132** |
| **41** | **26 or 40 [ MRI + CT GENERAL ]** | **992998** |
| 42 | cadaver/ or postmortem changes/ | 45988 |
| 43 | forensic sciences/ or forensic medicine/ or autopsy/ or forensic pathology/ | 65895 |
| 44 | atlases as topic/ or anatomy, artistic/ or exp anatomy, cross-sectional/ | 4347 |
| 45 | biological specimen banks/ or tissue banks/ | 7139 |
| 46 | specimen handling/ or dissection/ | 37661 |
| 47 | morphogenesis/ | 24942 |
| 48 | (postmort* or post-mort* or autop* or preautop* or cadaver* or forensic* or necrop* or obduction* or corps* or dead bod* or body remains or virtop* or ex-vivo or ((virtual or non-invasiv* or non-invasiv* or nondestruct* or non-destruct*) adj2 (pathol* or histol* or atlas* or anatom*))).tw,kf. | 369502 |
| 49 | ((atlas* and ((spat* adj3 (tempor* or map*)) or spatiotemp* or week-by-week)) or ((longitudinal or embryo* or f?et*) adj3 atlas*)).tw,kf. | 403 |
| 50 | ((f?etal or f?etus* or embryo* or brain) adj3 (specimen* or (samples not blood sampl*))).tw,kf. | 9562 |
| 51 | (((embryo* or f?etus*) adj3 collection*) or ((f?etuses or embryos) adj3 collect*)).tw,kf. | 1973 |
| 52 | ((macro* or microsc* or f?etal or f?etus* or brain or cranial or embryo* or pathol* or specimen* or tissue* or anatom* or histol* or virtual) adj3 dissect*).tw,kf. | 12685 |
| 53 | (morphogen* not (bone morphogen* adj3 protein*)).tw,kf. | 48448 |
| 54 | human f?etal development*.tw,kf. | 245 |
| **55** | **or/42-54 [ post-mortem ]** | **531734** |
| **56** | **41 and 55 [ III MRI/CT general + PM ]** | **32081** |
| **57** | **23 or 36 or 56 [ I II III muCT; UHF-MRI; PM CT/MRI ]** | **65232** |
| **58** | **18 and 57 [ fetus + I II III ]** | **977** |
| **59** | **remove duplicates from 58 [ deduplicate fetus + I II III ]** | **974** |

Appendix B Embase search strategy

Database(s): **Embase Classic+Embase**1947 to 2019 July 19 
Search Strategy: **2019-07-22**

| **#** | **Searches** | **Results** |
| --- | --- | --- |
| 1 | "fetus (anatomy)"/ or fetus bladder/ or fetus brain/ or fetus heart/ or fetus kidney/ or fetus liver/ or fetus lung/ or "embryo (anatomy)"/ or human embryo/ or fetus/ | 232372 |
| 2 | fetus death/ or stillbirth/ or fetus wastage/ or second trimester abortion/ or spontaneous abortion/ or pregnancy termination/ or induced abortion/ | 112689 |
| 3 | embryo development/ or fetus development/ | 110047 |
| 4 | f?etus*.tw,kw. | 161155 |
| 5 | (f?etal not (f?etal adj3 (blood or sera or serum or osteoblas* or osteocyt* or cord or cells))).tw,kw. | 294827 |
| 6 | (abortion* or stillbirth* or still-birth* or miscarriag* or early pregnancy loss* or (pregnancy adj2 terminat*)).tw,kw. | 120464 |
| 7 | ((human or abort* or normal) adj3 (embryo or embryos)).tw,kw. | 16813 |
| 8 | ((human or normal) adj6 ((embry* adj3 develop*) or embryogenes* or embryo-genes*)).tw,kw. | 6974 |
| 9 | ((early or prenat* or pre-nat* or trimester*) adj2 (developmen* or developing) adj2 (human or embry* or brain* or cereb*)).tw. | 9173 |
| 10 | (neuroembryo* or neuro-embryo*).tw,kw. | 194 |
| **11** | **or/1-10 [ FETUS ]** | **664993** |
| 12 | ((animal/ or animal experiment/ or exp animal model/ or nonhuman/ or exp female animal/ or exp veterinary medicine/ or (animal* or veterin*).jw.) not human/) or ((nonhuman or experimental model* or animal* or primat* or monkey* or ape or apes or veterinar* or ruminant* or cattle or bovine or cow or cows or horse or horses or mares or equine or sheep or ovine or lamb or lambs or goat* or pig or pigs or piglet* or sow or sows or sus scrofa or swine or swines or porcine or pup or pups or dog or dogs or canine or bitch* or cat or cats or feline or rodent* or lagomorph* or rabbit* or hare or hares or pikas or rat or rats or mice or mouse or murine or serpent* or python*) not human*).ti. or ((knockout* or knock-out* or transgenic* or genetic* modif* or antler* or snake or snakes or chick* or chicken* or shark or sharks or gecko*) not (human or humans)).tw,kw. [animal filter] | 7029596 |
| 13 | exp transplantation/ or exp cell therapy/ or (transpl* or allotranspl* or heterotranspl* or homotranspl* or autotranspl* or graft* or allograft* or heterograft* or homograft* or autograft* or cell-therap*).tw,kw. [transplantation] | 1362251 |
| 14 | (cells or in-vitro or cultur*).ti. | 1633084 |
| **15** | **or/12-14 [ animal/transplantation/cell culture exclusion filter ]** | **9070057** |
| **16** | **11 not 15 [ human fetus/embryology/development ]** | **444723** |
| **17** | **limit 16 to yr="1995 -Current"** | **283358** |
| 18 | micro-computed tomography/ or (iodination/ and computed tomography scanner/) | 18420 |
| 19 | (microCT* or muCT* or mu-CT* or ((micro or microcomp* or microfocus) adj6 (tomogra* or CT or CTs)) or (microscop* adj (tomogra* or CT*)) or microtomogra* or mutomogra* or mu-tomogra*).tw,kw. | 25300 |
| 20 | (diceCT or dice-CT or (diffus*-Iodin* adj2 contrast-enhanc* adj2 (computed tomograph* or CT or CTs))).tw,kw. | 28 |
| 21 | (PMCT or PMCTs or PM-CT or PM-CTs or ((postmort* or post-mort* or autop* or virtop* or corps* or necrop* or ex-vivo) adj6 (tomogra* or CT or CTs))).tw,kw. | 4219 |
| **22** | **or/18-21 [ I muCT ]** | **31855** |
| 23 | nuclear magnetic resonance/ or nuclear magnetic resonance imaging/ or diffusion tensor imaging/ or diffusion weighted imaging/ or whole body mri/ or nuclear magnetic resonance scanner/ or tractography/ | 920058 |
| 24 | (ultra-high field* or high field nuclear* or high field magnetic* or high angular resolution diffus*).ct. | 172 |
| 25 | (MRI or MRIs or (magnetic adj2 imag*) or ((MR or NMR or MRM) adj3 (scan* or imag*)) or UHFMRI* or muMRI* or microMRI* or DTMRI or DTI or DWI or diffusion tension imag* or tractograph* or PMMRI* or PMFMRI* or HARDI).tw,kw. | 617838 |
| **26** | **or/23-25 [ MRI-general ]** | **1026658** |
| 27 | (ultra-high field* or high field nuclear* or high field magnetic* or high angular resolution diffus*).ct. | 172 |
| 28 | ((("7" or 7?0* or 7?1 or "8" or 8?0 or "9" or 9?0 or 9?4 or "10" or "11" or "12") adj1 (T or Tesla)) or 7T or 7?0T or 8T or 8?0T or 9T or 9?0T or 9?4T or 11T or 12T or 7TMR or 7?0TMR or 8TMR or 8?0TMR or 9TMT or 9?0TMR or 9?4TMR or 11TMR or 12TMR).tw,kw. | 39042 |
| 29 | (((ultrahigh* or ultra-high*) adj2 field*) or UHF*).tw,kw. | 2960 |
| 30 | (high adj2 field* adj9 (MRI or MRIs or (magnetic adj2 imag*) or ((MR or NMR) adj3 (scan* or imag*)) or DTMRI or DTI or DWI or diffusion tension imag* or tractograph* or PMMRI* or PMFMRI*)).tw,kw. | 3919 |
| 31 | ((high or ultrahigh) adj2 (resol* or strength*) adj3 (MRI or MRIs or (magnetic adj2 imag*) or ((MR or NMR) adj3 (scan* or imag*)) or DTMRI or DTI or DWI or diffusion tension imag* or diffusion imag* or tractograph*)).tw,kw. | 10147 |
| 32 | (super-resolution adj (f?et* or embryo*) adj3 (MRI* or (magnetic adj2 imag*) or (MR adj3 (scan* or imag*)) or DTMRI or DTI or DWI or diffusion tension imag* or tractograph*)).tw,kw. | 1 |
| 33 | (microMR* or muMR* or ((micro or microcomp* or microfocus or microscop* or mu) adj3 (MR or MRI* or magnetic resonan* or NMR or MMR))).tw,kw. | 3427 |
| 34 | (PMMRI* or PMFMRI* or PMMR or ((postmort or post-mort*) adj (scan* or imag*)) or ((PM or PMF) adj3 (MRI or MRIs or resonance imag* or MR or MRM)) or ex-vivo-MRM).tw,kw. | 694 |
| 35 | ((Bruker adj3 (Biospin* or scan* or MR or MRI)) or BioMR-70-20 or PharmaScan* or SIS 200-330).mp. | 1762 |
| 36 | or/27-35 [ UHF or PM MRI ] | 56511 |
| **37** | **26 and 36 [ II UHF/micro MRI ]** | **24314** |
| 38 | tomography/ or *computer assisted tomography/ or high resolution computer tomography/ or whole body ct/ or x-ray computed tomography/ or computed tomography scanner/ | 212517 |
| 39 | ((CAT-scan* or (comput* adj3 tomogra*)) not (angiogra* or MDCT* or MSCT* or multislic* or multidetect* or dual-energ* or DECT)).tw,kw. | 311739 |
| 40 | ((CT or CTs) adj6 (scan* or imag* or MRI* or tomogra* or whole body or 3D or 3-D)).tw,kw. | 330903 |
| **41** | **or/38-40 [ CT GENERAL ]** | **605222** |
| **42** | **26 or 41 [ MRI + CT GENERAL ]** | **1495841** |
| 43 | exp cadaver/ or postmortem change/ | 54841 |
| 44 | forensic medicine/ or forensic science/ or forensic pathology/ | 47434 |
| 45 | autopsy/ | 189047 |
| 46 | ex-vivo-study/ | 64443 |
| 47 | morphogenesis/ | 39938 |
| 48 | (postmort* or post-mort* or autop* or preautop* or cadaver* or forensic* or necrop* or obduction* or corps* or dead bod* or body remains or virtop* or ex-vivo or ((virtual or non-invasiv* or non-invasiv* or nondestruct* or non-destruct*) adj2 (pathol* or histol* or atlas* or anatom*))).tw,kw. | 539076 |
| 49 | ((atlas* and ((spat* adj3 (tempor* or map*)) or spatiotemp* or week-by-week)) or ((longitudinal or embryo* or f?et*) adj3 atlas*)).tw,kw. | 537 |
| 50 | ((f?etal or f?etus* or embryo* or brain) adj3 (specimen* or (samples not blood sampl*))).tw,kw. | 13731 |
| 51 | (((embryo* or f?etus*) adj3 collection*) or ((f?etuses or embryos) adj3 collect*)).tw,kw. | 2579 |
| 52 | ((macro* or microsc* or f?etal or f?etus* or brain or cranial or embryo* or pathol* or specimen* or tissue* or anatom* or histol* or virtual) adj3 dissect*).tw,kw. | 18961 |
| 53 | (morphogen* not (bone morphogen* adj3 protein*)).tw,kw. | 57545 |
| 54 | human f?etal development*.tw,kw. | 314 |
| **55** | **or/43-54 [ post-mortem ]** | **737884** |
| **56** | **42 and 55 [ III MRI/CT general + PM ]** | **45639** |
| **57** | **22 or 37 or 56 [ I II III muCT; UHF-MRI; PM CT/MRI ]** | **94146** |
| **58** | **17 and 57 [ fetus + I II III ]** | **1646** |
| **59** | **remove duplicates from 58 [ deduplicate fetus + I II III ]** | **1623** |
| **60** | **59 not medline.cr. [ deduplicate fetus + I II III - embase records only ]** | **1483** |
