# Supplementary figures and images for: Novel imaging techniques to study postmortem human fetal anatomy: a systematic review on microfocus-CT and ultra-high-field MRI
Source: Eur Radiol. 2019 Dec 13;30(4):2280–92. doi: 10.1007/s00330-019-06543-8 (PMC7062658; doi:10.1007/s00330-019-06543-8)

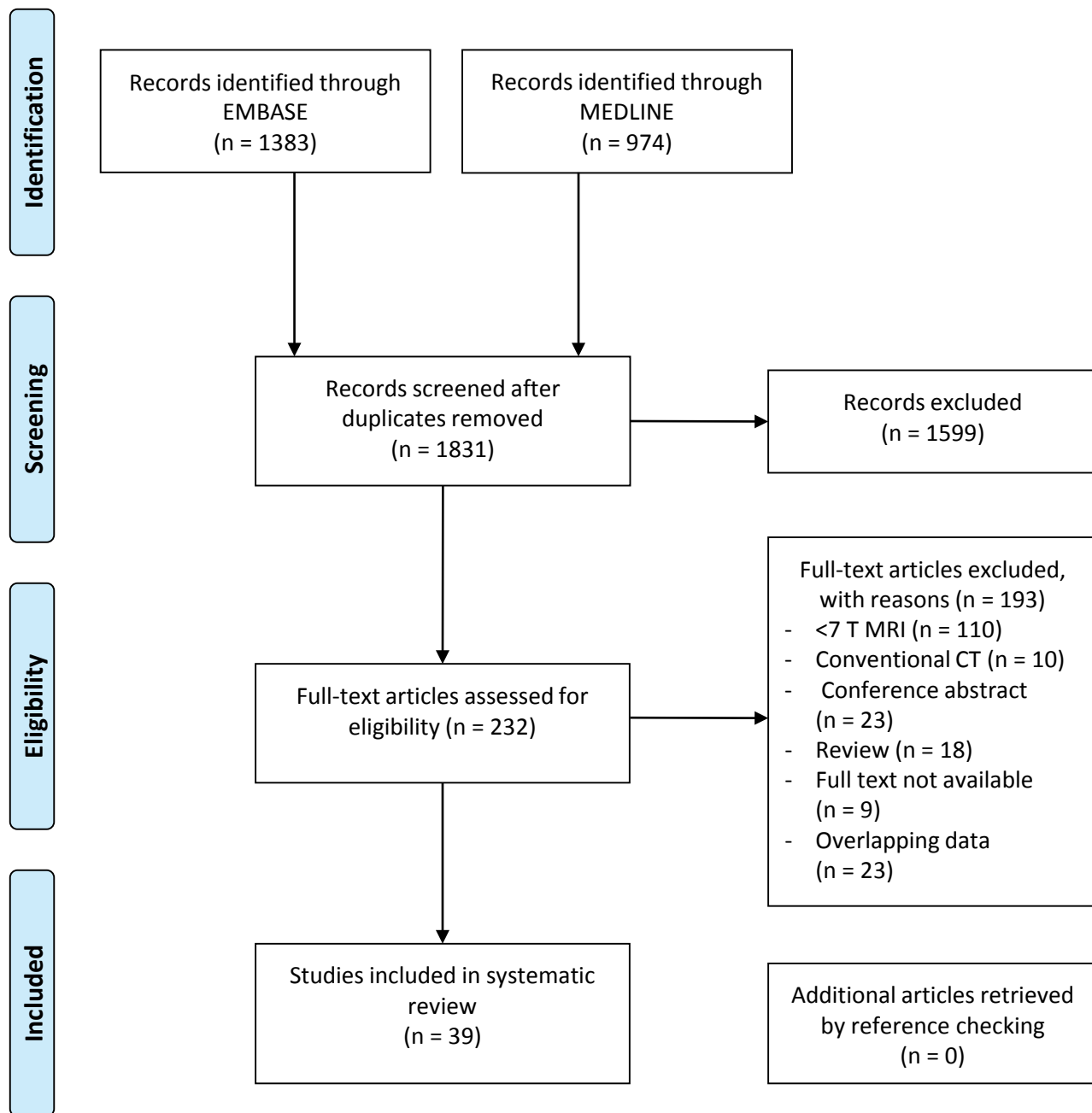

Supplement: Supplementary file 3 — (PDF 76 kb) [file 330_2019_6543_MOESM3_ESM.pdf]
