# Supplementary material for: Novel imaging techniques to study postmortem human fetal anatomy: a systematic review on microfocus-CT and ultra-high-field MRI
Source: Eur Radiol. 2019 Dec 13;30(4):2280–92. doi: 10.1007/s00330-019-06543-8 (PMC7062658; doi:10.1007/s00330-019-06543-8)
Supplement: Supplementary file 4 — (PDF 72 kb) [file 330_2019_6543_MOESM4_ESM.pdf]

Staining

Background

The success of staining is mainly dependent on three factors: 1) specimen size, 2) staining solution concentration and 3) staining time. In larger specimens the staining fluid has to penetrate deeper to reach the core of the fetal body. Staining concentration and time are interdependent factors. A higher concentration leads to a higher osmotic pressure, resulting in faster diffusion of the staining solution, enabling shorter staining exposure times. However, extended exposure time (to ensure complete and even staining) with higher concentration can result in overstaining and loss of tissue differentiation and/or tissue shrinkage. See supplementary table 1 for the appropriate amounts of solvent and solutes needed to mix the appropriate Lugol's (potassium triiodide, I2K) concentration.

Authors	Target %	I2 (grams)*	KI (grams)*	Volume of deionized water (ml)	Total solution volume (ml)
Spaw and Witmer 2014	1.25	0.42	0.83	100	100
Lombardi et al. 2014	3.75	1.75	2.50	100	100
Spaw and Witmer 2014	5.5	1.83	3.67	100	100
Lombardi et al. 2014	7.5	3.50	7.50	100	100

Supplementary table 1. Lugol solutes and solvent ratios. *We use I2 and KI 99+% from Fischer Scientific in 3.75% concentration.

Protocol

Grind the solid I2 using a mortar and pestle for quicker disolvement. Put the I2 and KI in a glass erlenmeyer and add an appropriate amount of deionized water. For convenience use magnetic stirrer. The powder should be disolved in a couple of minutes, depending on the concentration and quantity of the solution. Always work under a fumehood and store the Lugol's solution in the dark.

Immerse the specimen in Lugol for at least 2 days depending on specimen size. If possible, use conventional CT scanning to confirm that the Lugol has penetrated the specimen well enough.

Destaining

Background

Further histological examination and gross dissection of fetal specimens remains possible after staining when using an iodine-based staining (e.g. Lugol). Destaining is necessary as the staining solution gives the specimen a red-brown color. Destaining is fairly quick and easy, by completely submerging the specimen in a 4 to 5% weight/volume (w/v) sodium thiosulfate solution for hours to days (see Supplemental Digital Content 3 for formulations and protocols). It should be noted however, that destaining does not restore a specimen to its original chemical state. Rather than being deiodinated, sodium thiosulfate reacts with aqueous triiodide reducing it to iodide. Unlike aqueous triiodide, which is red-brown, iodide is transparent and remains in the specimen after destaining. If necessary, withdrawal of the iodide is possible by with leaching; clean water or fresh storage solution is used to displace iodide due to an osmotic imbalance. However, this process often takes weeks and requires frequent refreshment of the leaching solution, as it becomes saturated with iodide. See supplementary table 2 for the appropriate amounts of solvent and solute needed to mix the appropriate sodium thiosulfate concentration.

Target %	Sodium thiosulfate* (grams)	Volume of deionized water (ml)	Total solution volume (ml)
4	4	100	100
5	5	100	100

Supplementary table 2. Sodium thiosulfate pentahydrate concentrations. *We use sodium thiosulfate pentahydrate >99.5% from Sigma-Aldrich in 4% concentration.

Protocol

Dissolve the sodium thiosulfate in deionized water. The crystals easily dissolve in a couple of minutes. Completely submerging the specimen into the destaining solution for hours to days depending on the size of the specimen. It is recommended to always have a stock ready to destain samples and clean stain solution spills.

NOTE: Destaining does not restore a specimen to its original chemical state.
